# Family Planning Practice by Patterns of Marriage in the North of Iran

**Published:** 2017-01

**Authors:** Ziba TAGHIZADEH, Abouali VEDADHIR, Fatemeh BAYANI, Fereshteh BEHMANESH, Abbas EBADI, Abolghasem POURREZA, Mohammad Jalal ABBASI-SHAVAZI, Ali BIJANI

**Affiliations:** 1. Dept. of Midwifery and Reproductive Health, School of Nursing and Midwifery, Tehran University of Medical Sciences, Tehran, Iran; 2. Dept. of Anthropology, Faculty of Social Sciences, University of Tehran, Tehran, Iran; 3. Honorary Research Associate (HRA), UCL Department of Science and Technology Studies (STS), University College London, Gower Street, London, WC1E 6BT, United Kingdom; 4. Social Determinants of Health Research Center, Babol University of Medical Sciences, Babol, Iran; 5. Dept. of Midwifery, Faculty of Medicine, Babol University of Medical Sciences, Tehran, Iran; 6. Behavioral Sciences Research Center (BSRC), Nursing Faculty of Baqhiyatallah University of Medical Sciences, Tehran, Iran; 7. Dept. of Health Management and Economics, School of Public Health, Tehran University of Medical Sciences, Tehran, Iran; 8. Dept. of Demography, Faculty of Social Sciences, University of Tehran, Tehran, Iran; 9. Australian Demographic and Social Research Institute (ADSRI), Australian National University, Canberra, Australia

**Keywords:** Patterns of marriage, Family planning, Reproductive behavior

## Abstract

**Background:**

The fertility experience in Iran suggests that the family planning programs had an effective role in the fertility reduction. This study aimed to specify patterns of marriage in Iran and especially in a northern city of Iran and to investigate the association between patterns of marriage and contraceptive use before first pregnancy and current contraceptive use.

**Methods:**

In this cross-sectional study, following the implementation of an expert panel in order to investigate marriage patterns, 880 women aged 15–49 yr old, were selected by multistage cluster sampling and completed the “reproductive practices” questionnaire in Babol City, northern Iran, in 2013. The data were analyzed using IBM SPSS ver.16 and descriptive and analytical parameters.

**Results:**

There are three patterns of marriage in the northern part of Iran: Traditional, Mixed and Modern marriage and between different patterns there is no statistically significant difference in the contraceptive use.

**Conclusion:**

According to the lack of significant relationship between patterns of marriage and the contraceptives use, which is one of the proximate determinants of fertility, the policy makers should pay attention to other determinants of fertility in order to manage the problems and implications of population decline in the country.

## Introduction

Significant changes in various aspects of the family are taking place, for example, the rapid decline in fertility during the years 1985 to 2000 from 6 children to 2 children, increasing the education level of women, the time of marriage and family formation process. One of these changes is related to the patterns of marriage. The attitudes about patterns of marriage are changing (1, 2). In the past, parents or a member of the family usually selected the spouse. But todays, the patterns of marriage have changed from the traditional way to the modern choice by couples themselves (3–9). The fading traditional marriage system in Southeast Asia reflects a deep level of abdication of power by the parents (10, 11). Traditional marriages have taken place at an early age and spouses were selected from relatives; but, modern marriages often happen at an older age and with the low age difference between couples (12).

Several studies have examined the consequences of the marriage process (1, 13, 14). One of the consequences is the fertility and related factors, such as the use of contraceptives. Contraception methods are proximate determinants of fertility and, in recent years, using these methods was an important factor in the decline in birth rates (13, 15).

During the final decades, throughout Europe, the fall in the birth rate resulted in the invention of a new term, the Lowest-low fertility rate of 1.3 TFR (16) or Ultra-low fertility rate of 1 TFR (17) and predicts a significant decrease in the population of Europe (16). Hence, over the past few decades, the concern about rapid population growth and high fertility has turned the population decline to under the sub-replacement fertility levels. Currently, nearly half of the population in countries have low fertility or are below sub-replacement fertility levels. By the middle of the 21st century, three-quarters of developing countries will also reach this sub-replacement fertility level or be below this level (18, 19).

Many factors such as the government’s commitment to the family planning program and the support of the country’s religious leadership, decline in infant mortality, economic pressures placed on families, increased opportunities for education and employment for women may be incentives for couples to have fewer children. An increase in educational opportunities for women may also be associated with greater use of contraceptives (20).

However, the beginning of the decline of fertility in developing countries was after 1965, when better and more modern methods of contraception, such as IUD, various pills and newer methods of sterilization became common and abortion became legal (21). Now, 62% of women 14–45 yr old worldwide, use one of the contraceptive methods (22). In Iran, the first family planning policy was implemented in 1967. The family planning program targeted urban women, rather than providing training to their husbands. For this reason, and also because of the lack of financial and technical resources, this program had limited success (23). After the revolution, family planning program began with the goal of reducing the total fertility rate to 2.3 children per mother from 1981 to 2010 (24, 25). However, the family planning program was realized a decade sooner, so that in 2000, Iran was very close to replacement fertility levels (2.2 children per woman) (26) and in 2009 this was 1.8 (27). The presupposition of this study is based on the theory of motivated action established by Bandura. According to this theory, motivated action with a sense of self-efficacy toward a desired outcome includes volition without feeling coercion. One potentially important component of the volition is marriage volition. “Volition in marriage” can be effective in the use of the contraceptive as a result of better relationship between husband and wife (12).

Now, the age pyramid of Iran is in transition from youth to old population and it has been predicted that the Iranian population will be quite old by the year 2050. As a result, the continuity of fertility below the replacement level and increased mortality due to aging population may have both negative population growth and a reduction in workforce, which can affect economic growth and development (24, 26).

The experience of fertility transition in Iran indicates that it was proportionate to the changes occurred in various economic, social and some traditional family aspects, leading to the related changes in patterns of marriage and, ultimately, changes in behavior, ideals and aspirations of the people for childbearing (24, 26, 28).

The dramatic reduction of traditional marriage may have a significant impact on the increased use of contraceptives to limit fertility. Demographers believe that this revolution in the way of marriage will determine the time of first birth (14). Having the power to choose the spouse is associated with child number, intention to use contraceptives and the time between marriage and the first use of birth control methods (12). In addition, one of the reasons for reducing the interval between marriage and first pregnancy is the increased frequency of intercourse and this increase represents a shift from traditional to modern marriage. Having a better knowledge of each other, initial intimacy and physical attractiveness along with love matches result in increased frequency of intercourse and thus can increase the chances of pregnancy (29).

Realizing the demographic problems requires the examination of facts and reasons. Since one of the main priorities of developing countries is demographic studies, in particular, studies related to fertility, family planning programs, and the marriage process because of their important role in increasing or decreasing population growth.

This study aimed to 1) specify patterns of marriage in Iran and especially in a northern city of Iran and, 2) investigate the association between patterns of marriage and contraceptive use before first pregnancy and current contraceptive use.

## Materials and Methods

### Study design

In this cross-sectional study, after receiving permission from the Ethics Committee of Tehran University of Medical Sciences on 22 Mar 2013 (Ref. 23263-1297), an extensive literature review and several meetings were conducted by the research team to determine the patterns of marriage in Iran. The draft was prepared for the patterns of marriage and a panel of experts meeting was held, attended by 11 experts to finalize these patterns of marriage. The items related to patterns of marriage have been set in this way. Then, the researcher-made questionnaire of “reproductive behaviors” was used to determine the fertility practices of women. The validity and reliability of the tools (women’s reproductive behaviors in patterns of marriage) were examined.

For psychometric assessment of the tool, the content validity and face validity (qualitative and quantitative methods) were performed. Test-re-Test with 20 individuals within 2 wk and examined correlations were used to assess reliability. Finally, a pilot study with 80 subjects was conducted.

### Setting

This research is part of a larger study on the reproductive behaviors of married women aged 15 to 49 who were living in rural and urban areas in four geographic areas of six districts of Babol City. This study began in Apr 2013 and completed in Jan 2014.

### Participants

After completing psychometric assessment of tool, interviewers in medical health centers selected 880 eligible women by multistage cluster sampling. The Inclusion criteria of this study was: married women aged 15 to 49; resident of Babol; having at least one child and with no diseases regarding reproduction such as the primary and secondary infertility; a kind of disease that can cause interference in their fertility; and no diagnosed severe mental disability and psychiatric disorders which may cause inability to respond to the questionnaire. The exclusion criteria of this study were the lack of response to a maximum 10% of the questions (30).

### Study bias

In older women, there was probability of recall bias. Due to the randomness of the sampling and the presence of all age groups in all three patterns of marriage, this bias appears not to change because of the study.

### Statistical methods

After completing the questionnaire, data were analyzed using IBM SPSS ver.16 and descriptive and analytical parameters, Kruskal-Wallis, Chi-square and ANOVA Table were used to examine the relationship between patterns of marriage and contraceptive use before first pregnancy and current contraceptive use.

## Results

The data for 880 eligible women respondents were analyzed. Summarizing the views of the expert panel, eight items related to patterns of marriage were designed. According to the suggestions of expert panel and offered definitions of traditional and modern marriage from different sources (14, 31), 8 patterns of marriage, based on the mate selection by spouses or parents, friendship, and engagement before marriage and during marriage satisfaction have been integrated in 3 patterns of the traditional, modern and mixed. Mixed patterns of marriage are common in Iran. In this model, they marry without friendship before marriage, but with the consent of both spouses. The mixed pattern of marriage had the highest frequency (77.2%); the modern pattern of marriage (11.1%) with very little difference from the traditional pattern of marriage (11.7%) had the lowest frequency (Table 1). The comparison of social- demographic information about women in three different patterns of marriage is given in Table 2.

**Table 1:** Marriage patterns of the participated married women (15–49 yr) in Babol, Iran (first division)

** Pattern of Marriage **	** Frequency **	** % **
** 1.Traditional **	103	11.7
I did not know my husband before marriage, we met each other through matchmaking and I was not really satisfied and I was somehow forced to marry him.	79	9
I knew my husband before marriage and through matchmaking and somehow with no satisfaction, I was really forced to marry him.	21	2.4
Our marriage was a sort of arranged marriage.	3	0.3
2 ** .Mixed **	679	77.2
I knew my husband before marriage and I was married through matchmaking and with the approval and satisfaction of myself and my family.	212	24.1
We did not know each other before marriage. I met him through matchmaking and we married with my consent and my family’s approval.	467	53.1
** 3.Modern **	98	11.1
We were intrigued and wanted each other before marriage and married with our family’s approval.	72	8.2
We were intrigued, wanted each other before marriage, and married despite the objections of our families.	24	2.7
Even though we were friends before our marriage, I was not going to marry him and I was actually rather forced to marry him.	2	0.2
4. Please note other patterns.	0	0
Total	880	100

**Table 2:** Socio-demographic characteristics of married women (15–49 yr) in three marriage patterns in Babol

** Marriage patterns **	** Mixed **	** Traditional **	** Modern **	** Value **	***P* -value **
** Variables **	** Frequency (%) **	** Frequency (%) **	** Frequency (%) **		
** Level of education **					
Illiterate	24 (2.7)	11(1.3)	3(0.3)		
Primary school	111 (12.6)	23(2.6)	15(1.7)		
Guidance school	121 (13.8)	16(1.8)	15(1.7)	21.44	0.04
High School and national diploma	245 (27.9)	38(4.3)	40(4.6)		
Bachelor	157 (17.9)	13(1.5)	20(2.3)		
Master’s degree or higher	20 (2.3)	2(0.2)	5(0.6)		
** Place of Birth **					
City	273(31.0)	30(3.4)	46(5.2)	7.03	0.03
Village	406(46.1)	73(8.3)	52(5.9)		
** Residence **					
City	382(43.4)	46(5.2)	57(6.5)	5.27	0.07
Village	297(33.8)	57(6.5)	41(4.7)		
** Employment status before the first pregnancy **					
Unemployed	525(59.7)	78(8.9)	74(8.4)		
Farmer	8(0.9)	6(0.7)	2(0.2)		
Laborer	9(1.0)	2(0.2)	2(0.2)		
Employee	68(7.7)	7(0.8)	4(0.5)		
Freelance	39(4.4)	5(0.6)	12(1.4)		
Professionals (engineer, physician, judge)	4(0.5)	1(0.1)	0(0)	22.45	0.07
Manager	1(0.1)	0(0)	0(0)		
Student	25(2.8)	4(0.5)	4(0.5)		
** Current Employment status **					
Unemployed	484(55.0)	70(8.0)	71(8.1)		
Farmer	8(0.9)	3(0.3)	2(0.2)		
Laborer	15(1.7)	6(0.7)	3(0.3)		
Employee	76(8.6)	10(1.1)	7(0.8)		
Freelance	67(7.8)	12(1.4)	12(1.4)	10.60	0.71
Professionals (engineer, physician, judge)	7(0.8)	1(0.1)	1(0.1)		
Manager	1(0.1)	0(0)	0(0)		
Student	21(2.4)	1(0.1)	2(0.2)		
** The Socioeconomic Position **					
Low	129(14.7)	39(4.4)	16(1.8)		
Medium	417(47.5)	51(5.8)	66(7.5)	21.57	0.00
High	131(14.9)	13(1.5)	16(1.8)		

*P* -value is significant in level 0.05

The mean age of the subjects in the traditional group (36.23±8.13) was higher than modern group (33.32 ± 8.48) (*P*=0.01). However, the mean age of the mixed group (34.99 ± 7.81) had no significant difference from the two other groups. The mean age of the spouse of the traditional group (40.33 ± 8.96) (*P*=0.01) and mixed group (39.33 ± 8.37) was higher than modern group (37.01 ± 8.23) (*P*=0.006). The mean marriage age (19.42 ± 4.0), the mean of age difference between couples (4.24 ± 4.66) and current employment of subjects indicated no significant difference in the patterns of marriage.

Using a contraceptive method before the first pregnancy and its type, and current contraceptive use and its type, the timing of current contraception method and intention to continue the current contraception method were also examined in the three patterns of marriage, the results of which are shown in Table 3. As indicated in Table 3, no significant difference was observed between three patterns of marriage in contraceptive use before first pregnancy and current contraception use.

**Table 3:** The contraceptive use among married women (15–49 yr) in three marriage patterns in Babol

** Marriage pattern Variable **	** Mixed **	** Traditional **	** Modern **	** Total **	***P* -value **
** Variable **	** Frequency (%) **	** Frequency (%) **	** Frequency (%) **	** Frequency (%) **	
**Contraceptive use before first pregnancy **					
Yes	315 (35.9)	42 (4.8)	50 (5.7)	407 (46.4)	0.27
No	364 (41.5)	61 (6.9)	46 (5.2)	471 (53.6)	
Contraceptive method before first pregnancy					
DMPA	1 (0.2)	0 (0)	0 (0)	1 (0.2)	
OCP	39 (9.6)	2 (0.5)	7 (1.7)	48 (11.8)	0.34
Condom	61 (15)	4 (1)	6 (1.5)	71 (17.4)	
Withdrawal	215 (52.8)	35 (8.6)	37 (9.1)	287 (70.5)	
** Current Contraceptive use **					
Yes	608 (69.3)	91 (10.4)	84 (9.6)	783 (89.3)	0.77
No	70 (8)	12 (1.4)	12 (1.4)	94 (10.7)	
** Current contraceptive Method **					
TL	0.06113 (14.4)	12 (1.5)	10 (1.3)	135 (17.2)	
Vasectomy	23 (2.9)	3 (0.4)	4 (0.5)	30 (8.39)	
IUD	14 (1.8)	1 (0.1)	6 (0.8)	21 (7.29)	
DMPA	7 (0.9)	2 (0.3)	0 (0)	9 (1.1)	
OCP	83 (10.6)	14 (1.8)	10 (1.3)	107 (13.6)	
Condom	137 (17.5)	16 (2)	11 (1.4)	164 (20.9)	
Withdrawal	216 (27.6)	42 (5.4)	43 (5.5)	301 (38.4)	
Other methods	14 (1.8)	2 (0.3)	1 (0.1)	17 (2.2)	
** Intention continuation of current contraceptive method (Year) **					
> 1	32 (4)	8 (1)	4 (0.5)	44 (5.5)	
1–2	13(1.6)	4 (0.5)	1 (0.1)	18 (2.2)	
3–4	4 (0.5)	0 (0)	1 (0.1)	5 (0.6)	0.32
5–6	16 (2)	2 (0.2)	6 (0.7)	24 (3)	
I don’t know	178 (22.2)	23 (2.9)	27 (3.4)	228 (28.4)	
Until menopause	337 (46.9)	60 (7.5)	47 (5.9)	484 (60.3)	

*P*-value is significant in level 0.05

## Discussion

In this study, there was three patterns of marriage in the women of reproductive age in Babol city: traditional, modern and mixed marriage.

The use of contraceptive methods was investigated by three patterns of marriage and analysis demonstrated no statically difference between three groups. There are many factors that explain using or not using the family planning recommendations and contraceptive methods, including, the demographic, socio-cultural, economic and family-related variables (32).

Couples who speak together and have joint decisions to stop or delay childbearing were more likely to use contraceptives (33, 34). Family planning program evaluators and demographers define restrictions on communication functions as one of the factors limiting the impact of family planning programs (32). Changes in marital relationships are identified as an important factor in theories of fertility decline. Family planning or programming to determine the number of children and family size means to engage human volition in reducing family size and the population of the country. Women who are independent, autonomous and free to decide in their parent’s home, have this ability after marriage and are more able to talk about their reproductive matters to their husbands (12).

Communicative variables of couples such as decisions about family size and family planning and husband’s confirmation about contraceptive methods were observed all effective on the current use of effective contraceptive. Intimate communication of couples may be effective in the use of contraceptive methods in several ways: First, it helps change attitudes about the physical act of effective and consistent use of contraception. Relation to the desired family size may allow couples to limit fertility. Second, the relationship between the couples may reduce the psychological burden of contraceptives. Psychological burdens are social-psychological pressures, which arise due to incorrect judgments about contraceptive methods, can cause emotional stress and therefore may not be used. If one of the spouses has an open attitude about contraceptives and reinforces the belief that it is an acceptable social behavior, the intimate relationship may reduce the psychological burden. Third, communication and agreement between the spouses may be where a certain number of children known as norm also reduce the demand for children. Couple’s communication makes it possible to reach agreement on a plan to reduce the spacing between children, which may result in family size reduction (35).

Cognitive problems in the first three years of marital life were demonstrated the most difficult problems for couples. Fertility practice in marriages that took place with prior friendship varies compared to traditional marriage without previous friendship (36). Although evaluation of the independent relationship between marriage volition and fertility practice will require further studies (12), the present study investigated the effect of marriage type, which has been one of the division criteria based on the marriage volition on contraceptive use as a fertility practice affecting the population. There are three types of marriages in the northern part of Iran: Traditional marriage takes place with parents’ coordination and without the will of one of the individuals or both; modern marriage, associated with prior friendship and sometimes happens with or without parental consent; and mixed marriage, which is the most common type in Iran. Because friendship before marriage is considered taboo in Iran, in mixed marriage, couples, with or without previous familiarity, talk together during matchmaking or during several meetings, and then eventually marry with the consent of both parents. In addition, patterns of marriage have no effect on contraceptive use before first pregnancy and current contraceptive use.

However, couples with traditional marriage were less satisfied in their marital relationship, had restrictions in connection with their wives and have less decision-making power. More power to choose the spouse was correlated with better relationship between the couple for family planning and the use of modern contraceptive methods, the results of which were different with the present study. On the other hand, more familiarity and intimacy with love matches could increase frequency of intercourse and thus the chances of pregnancy. Perhaps the reason of differences between the findings of this study was that it has been conducted in Malaysia, Taiwan, Korea, and China, where the use of contraception after marriage and before the first pregnancy is low. But in Iran, the use of contraception is common after marriage, especially among educated people (29). One of the changes that occurred after the Iranian revolution is that women’s education has increased dramatically more than ever before, and concurrent with the government’s efforts to reduce the population, a substantial increase in contraceptive use has occurred. The family planning program of the government had a significant role (20, 37). For example, the Ministry of Health, Treatment and Medical Education has provided unlimited resources for family planning to married couples to strengthen the small family as norm, and to help couples to avoid unwanted pregnancy. For this reason, traditional marriage has been replaced by mixed and modern marriages in Iran and this change is one of the factors influencing the use of contraceptive methods (37). The results of the present study showed no difference in the contraceptive use. Probably, the major reasons are encouraging families to use modern contraceptive methods and to have fewer children in the health-medical centers, and economic problems of the society, particularly after the Iran-Iraq War. The public education has been implemented similarly for both rural and urban population and this resulted in the elimination of urban- rural gap infertility and the use of modern contraceptives (37, 38).

One limitation of this study is the lack of study of social network factors. The importance of communication was showed between peers or siblings on the formation of reproductive practice (20, 35, 39). Further research to identify the impact of social interaction on the reproductive practice is recommended.

## Conclusion

Marriage patterns are not significantly associated with the use of contraceptives. Most likely, other factors such as the level of education of women, economic problems, and the governmental family planning had a significant role in fertility reduction in Iran. Despite the changes in marriage patterns, other socio-economic changes occurred after the Iranian revolution including increases in women’s education and employment assimilates the reproductive practices of women, including the use of contraceptive methods. Therefore, authorities try to design scientific management methods and specific strategies in order to reduce the over-population problem, while addressing the economic problems by giving them information about the negative consequences of excessive reduction of fertility, instead of removing the free contraceptives items which may cause problems such as an increase in the unwanted pregnancies, unsafe abortions and reproductive loss for the poor and low-income families.

## Ethical considerations

Ethical issues (Including plagiarism, informed consent, misconduct, data fabrication and/or falsification, double publication and/or submission, redundancy, etc.) have been completely observed by the authors.
